# Temporal and spatial properties of vestibular signals for perception of self-motion

**DOI:** 10.3389/fneur.2023.1266513

**Published:** 2023-09-13

**Authors:** Bingyu Liu, Jiayu Shan, Yong Gu

**Affiliations:** ^1^Center for Excellence in Brain Science and Intelligence Technology, Institute of Neuroscience, International Center for Primate Brain Research, Chinese Academy of Sciences, Shanghai, China; ^2^University of Chinese Academy of Sciences, Beijing, China

**Keywords:** vestibular, optic flow, self-motion, heading, rotation–linear motion

## Abstract

It is well recognized that the vestibular system is involved in numerous important cognitive functions, including self-motion perception, spatial orientation, locomotion, and vector-based navigation, in addition to basic reflexes, such as oculomotor or body postural control. Consistent with this rationale, vestibular signals exist broadly in the brain, including several regions of the cerebral cortex, potentially allowing tight coordination with other sensory systems to improve the accuracy and precision of perception or action during self-motion. Recent neurophysiological studies in animal models based on single-cell resolution indicate that vestibular signals exhibit complex spatiotemporal dynamics, producing challenges in identifying their exact functions and how they are integrated with other modality signals. For example, vestibular and optic flow could provide congruent and incongruent signals regarding spatial tuning functions, reference frames, and temporal dynamics. Comprehensive studies, including behavioral tasks, neural recording across sensory and sensory-motor association areas, and causal link manipulations, have provided some insights into the neural mechanisms underlying multisensory self-motion perception.

## Introduction

Organisms have evolved various sensory systems, such as vision, hearing, smell, taste, touch, vestibular, and proprioceptive senses to detect changes in body states and the surrounding environment accurately. Among these, the vestibular system provides fundamental sensory signals from basic survival functions to complex cognitive abilities in humans and other animals. Although often overlooked, the vestibular system facilitates numerous functions, from basic reflexes, such as the vestibulo-ocular reflex and postural control, to higher-level cognitive processes, such as spatial navigation, spatial memory, and bodily self-consciousness.

Recent investigations into the cortical processing of vestibular inputs, especially with well-designed behavioral paradigms and *in vivo* electrophysiological recordings in awake, behaving nonhuman primates, have yielded important insights into the temporal and spatial properties of these signals and their potential functions. It has been shown that one critical ability enabled by the vestibular system is navigation, which allows organisms to explore the environment efficiently (1). Two strategies are commonly used during navigation. One is landmark-based navigation, which relies heavily on visual landmarks, yet environmental changes could confound this strategy when the cues become unreliable. The other strategy is vector-based navigation (or path integration), which involves vestibular signals for continuously updating one’s heading and position (2–4). Specifically, linear and angular vestibular signals originating from peripheral otolith and semicircular canal organs, respectively, are transmitted through the vestibular nuclei and thalamus to cortical areas and the hippocampal system for spatial perception. For example, it has been shown that the head direction cell network, which functions as a “compass” in navigation, receives critical vestibular inputs from canals via the anterodorsal thalamus (ADN) (5, 6). Moreover, lesioning the peripheral vestibular inputs severely disrupts the formation and stabilization of head direction cells and navigation ability (7–10), indicating the critical function of the vestibular system in spatial navigation.

Another important pathway for the projection of vestibular signals to the cerebral cortex is the ventral posterior lateral nucleus (VPL), which is involved in the perception of the instantaneous direction of one’s movements through space, i.e., heading perception (5, 11, 12). Studies in humans and nonhuman primates have demonstrated that vestibular signals are necessary for accurately judging heading direction, especially when visual cues are absent (13, 14). Patients with bilateral vestibular damage exhibit a severely impaired ability to perceive self-motion (15). In nonhuman primate studies, an intact vestibular system is essential for self-motion perception (16, 17). Specifically, macaques were first trained to judge small heading directions that deviated from an internal straight-ahead reference accurately, and bilateral or unilateral surgical ablation of the peripheral vestibular organs was then conducted. Subsequently, the macaques’ heading ability was severely affected shortly after labyrinthectomy, as reflected in a substantial increase in their psychophysical thresholds. The deficit is specific to the vestibular system because the animals’ visual discrimination ability largely remained unaffected. After a few months, the macaques’ heading ability based on the vestibular cue gradually recovered to some extent, potentially due to compensation from other sensory systems, such as somatosensory input. However, the vestibular psychophysical threshold ultimately reached a plateau much higher than that before the labyrinthectomy, indicating the fundamental importance of vestibular signals for heading perception. In addition to heading, rotational self-motion is also important in encoding the displacement of head or whole body in the environment. Indeed, recent studies have shown that during naturalistic stimuli, neurons in VPL encode the head velocity efficiently and unambiguously (18, 19).

**Figure 1:**
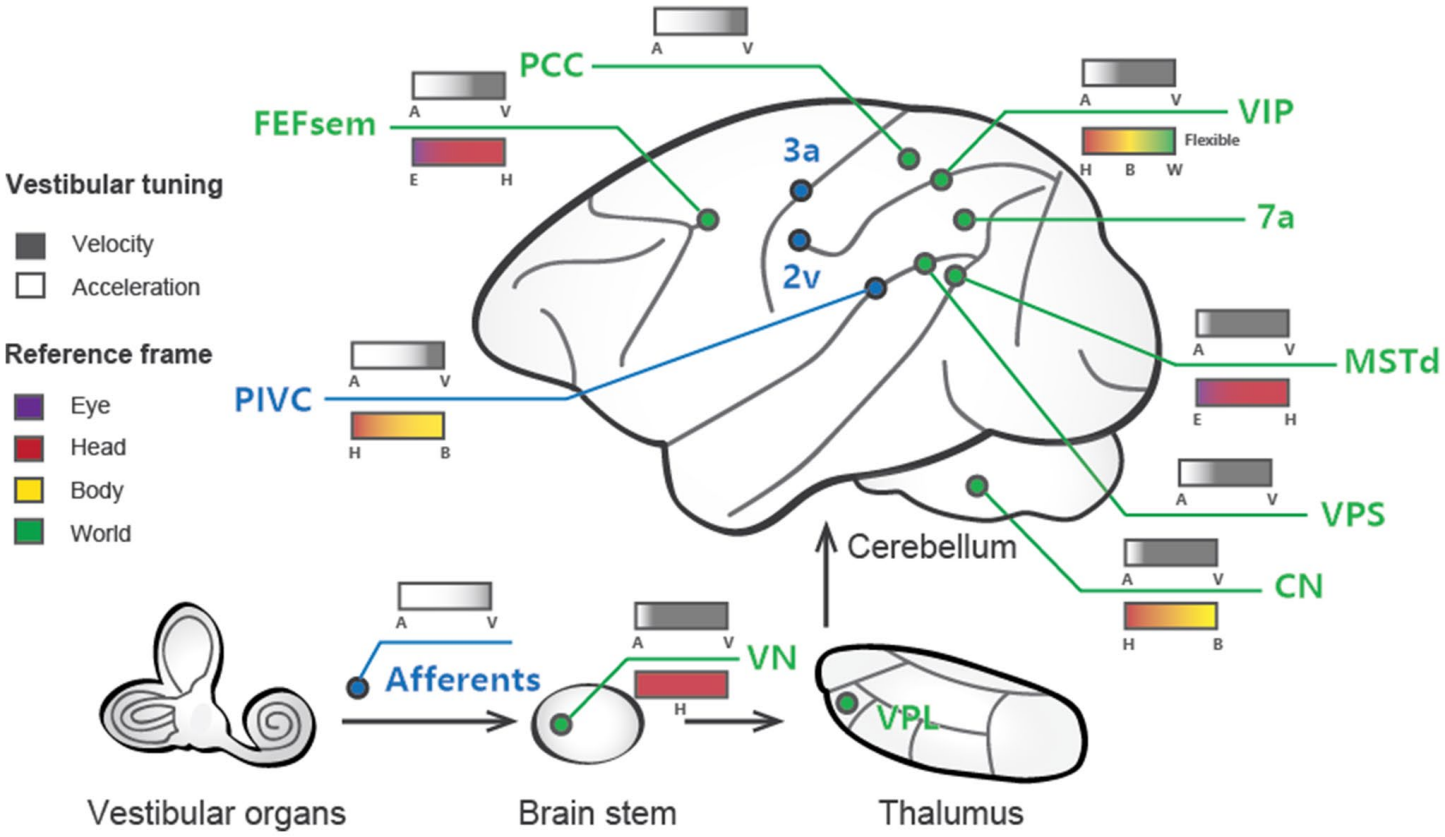
Schematic summary of vestibular signals broadly distributed across multiple regions in the brain. Cortical areas largely dominated by vestibular signals are labeled blue, while those that are multisensory (e.g., vestibular and visual) are shown in green. The vestibular tuning properties are depicted above the lines, with white denoting acceleration and black denoting velocity. The reference frames of the different areas are illustrated below the lines, with the varied colors signifying distinct reference frame preferences.

One interesting question is to what extent are vestibular signals represented in the brain, particularly in the cerebral cortex, which is thought to be more relevant to cognitive functions? Numerous methods have been adopted to address this question. In humans, caloric or galvanic stimulation techniques are used to activate the peripheral vestibular organs, while brain-wide activity is measured in functional magnetic resonance imaging (fMRI), magnetoencephalography (MEG), or positron emission tomography (PET) (20–22). In animals, one method is to electrically stimulate vestibular nerves through inserted microelectrodes while simultaneously recording evoked potentials in the cortex (23, 24). The aforementioned studies identified several cortical areas containing vestibular-related signals (Figure 1), indicating that such signals are broadly distributed in cortices and form a vestibular network (25). In addition to imaging and field potential measures, single-unit activity is also measured through electrophysiological techniques while the animals are physically rotated or translated through a motion platform (26–28). These studies thus provide data with high spatial (single cell) and temporal (millisecond) resolutions of vestibular activity, allowing us to look deeper into the neural correlates of vestibular functions.

Most of the regions in the cortex exhibiting vestibular signals also process other sensory information, such as visual, somatosensory, and proprioceptive information. There is probably no pure “vestibular” region in the brain. This implies that vestibular signals may be tightly associated with the other sensory channels to handle situations when self-motion is accompanied. Thus, much attention has also been paid to multisensory integration or interaction, an issue that will be further discussed below.

While much evidence has shown that vestibular information is essential for precise self-motion perception, signals from other sensory modalities are also important. The brain relies on other senses when one sensory input is ambiguous or absent in an ever-changing environment. Individual senses, such as vestibular or visual, could be noisy; thus, integrating multiple cues can reduce noise and increase strength in sensory representation. Regarding heading perception, numerous previous studies have confirmed that integrating vestibular and visual signals can enhance heading judgment precision, with a decrease in psychophysical thresholds in both human and nonhuman primates (29–31). Researchers have also explored the underlying mechanisms and found that vestibular and visual signals in many brain areas differ in their spatial and temporal properties (32, 33), raising the question of how different signals are integrated into the brain to enable accurate and precise self-motion judgments.

In the last two decades, extensive investigations have shed light on specific regions of the brain that play pivotal roles in perceiving self-motion and integrating information from the vestibular and visual systems. We have previously summarized the findings in a series of reviews. For example, some earlier reviews focus on vestibular coding of self-motion signals [For reviews, see Cheng and Gu (11), which presents vestibular signals in the perception of translation, curve motion and distance in details; (12), which primarily introduces tuning properties in self-motion areas; (34), which focuses on multisensory integration]. Compared to the previous reviews, here we summarize the current understanding of the tuning properties of vestibular signals in these areas during self-motion and discusses the outstanding questions regarding how the brain integrates vestibular and visual cues to perceive heading direction. We mainly focus on electrophysiological findings in nonhuman primates given the superior spatiotemporal resolution compared to human neuroimaging. The following questions are discussed: (1) the temporal and spatial dynamics of vestibular signals for self-motion perception across brain areas according to experimental and modeling studies, (2) the difference between vestibular and visual signals in self-motion and how these signals are utilized to make precise heading judgments, and (3) the mechanisms by which the brain may integrate vestibular and visual information despite their divergent features.

### Temporal and spatial tuning properties of vestibular signals

Investigations into vestibular processing in the cerebral cortex date back to 1949, when recordings were performed in cats (35). Early studies utilized electrical stimulation of the vestibular afferent nerves in anesthetized animals (mostly monkeys) to identify regions, such as areas 2v and 3a, that respond to vestibular input [Reviewed in Guldin and Grüsser (25)]. Subsequent single-unit recordings in awake animals during whole-body motion produced by spinning chairs, motion platforms, and centrifuges revealed cortical areas encoding vestibular information related to self-motion. Several such regions have been identified in macaque monkeys. To date, robust vestibular representations of self-motion have been found in numerous areas, including the dorsal medial superior temporal sulcus (MSTd) (28, 36, 37), the ventral intraparietal area (VIP) (38–42), the visual posterior sylvian area (VPS) (43), parietal insular vestibular cortex (PIVC) (27, 40, 44, 45), the smooth eye movement region of the frontal eye field (FEFsem) (46), area 7a (47), and the posterior cingulate cortex (PCC) (48). These areas constitute a network for vestibular processing in the brain [Figure 1; (11, 25, 49)].

**Figure 2:**
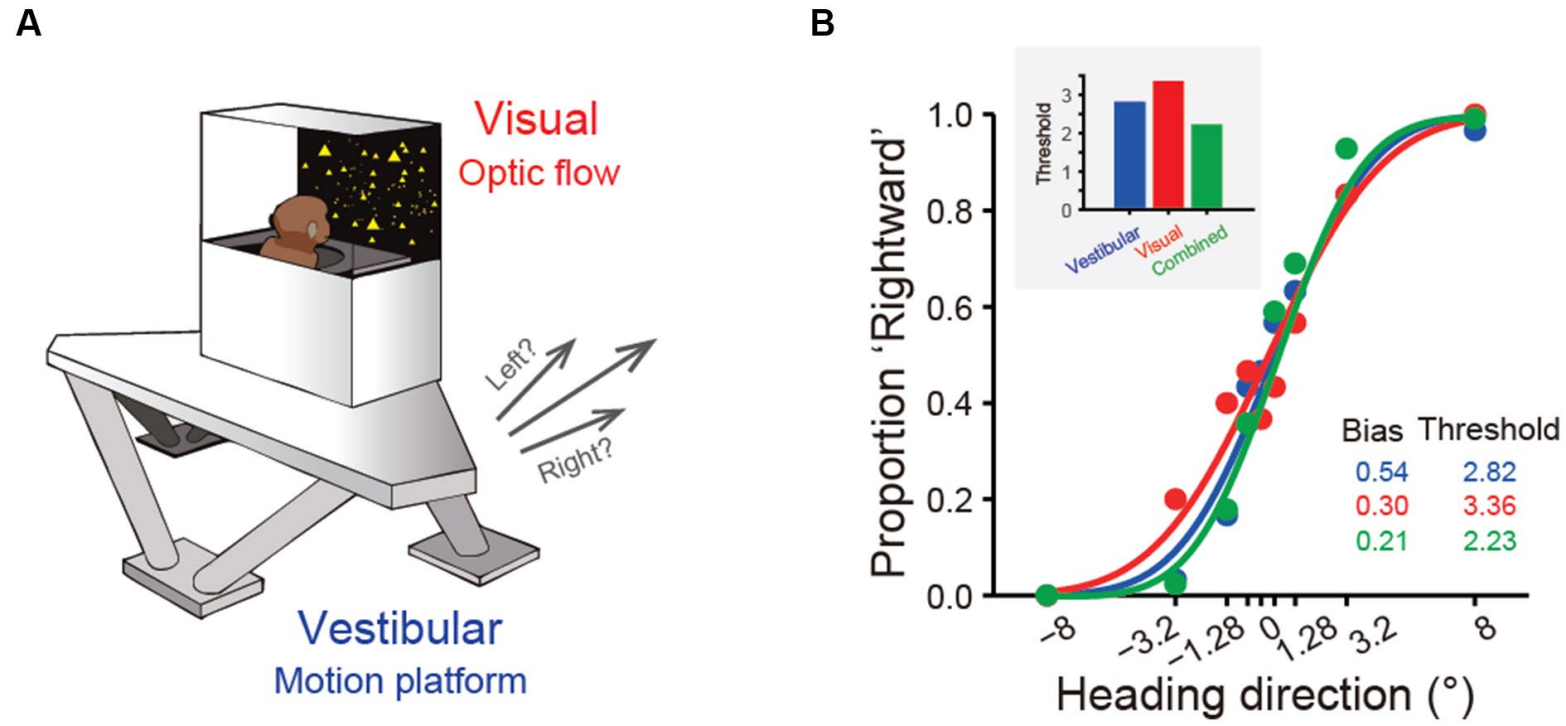
Experimental setup and near-optimal multisensory integration performance. **(A)** The virtual reality experimental setup of the heading discrimination task. Vestibular cues were provided by a 6-degree-of-freedom motion platform. A visual display is mounted on the platform, providing visual stimuli that simulate real motion. **(B)** Behavioral performance of one monkey in the vestibular-only, visual-only and visual-vestibular combined heading discrimination tasks. Note that the performance (threshold) of this monkey in the combined condition is near-optimal according to the prediction from the Bayesian integration theory (the inset figure).

Over the past two decades, more sophisticated experimental designs utilizing a 6-degree-of-freedom motion platform that can produce arbitrary movements in both translation and rotation in 3D space have enabled refined studies of the vestibular representations in these areas (Figure 2A). In these experiments, animals are passively moved in darkness to activate the vestibular system. These vestibular areas exhibit distinct response properties. The proportion of neurons responsive to translational vestibular stimulation is highest in the PIVC (76.4%), followed by the VPS (72.3%), FEFsem (71.87%), PCC (68%), MSTd (64%), VIP (49%), and 7a (40%) (28, 40, 44, 47, 48). Chen and et al. analyzed the temporal properties of vestibular signaling across areas to clarify the pathway of vestibular signals flowing in the cerebral cortex and found that the PIVC leads the response, followed by the VPS and VIP, with the MSTd exhibiting the longest latency to external stimuli (40). This result may suggest that vestibular information flows across areas, consistent with the notion that vestibular signals reach the PIVC first after thalamic relay from the vestibular nucleus (VN).

Vestibular signals originate in the peripheral organs that are sensitive to acceleration stimuli (50). Accordingly, electrophysiological recordings in vestibular afferents show predominantly acceleration coding, particularly during translation of the head or whole body in darkness (51). However, in the central nervous system, including the brainstem, cerebellum, and cerebral cortex, researchers have found that vestibular neurons exhibit much more plentiful temporal signals, including acceleration, velocity, jerk (a derivative of acceleration), and even position, reminiscent of the motor cortex (52). Specifically, experimenters first found that neurons in different regions exhibit single-and double-peaked responses, corresponding to velocity and acceleration components in the stimulus, respectively [(53, 54); also see review by Cheng and Gu (11)]. These findings suggest that progressive integration (e.g., velocity) and differentiation (e.g., jerk) of acceleration-dominant peripheral vestibular signals occur in the central nervous system and may serve distinct functions.

In addition to the varied temporal dynamics, the central vestibular signals have spatial selectivity. A widely used metric for quantifying the strength of directional tuning is the directional discrimination index (DDI) (55), which assesses the contrast in firing rates between the preferred and null directions of motion regarding the variability of the neuron’s response. Based on this measure, vestibular spatial tuning strength could be assessed and compared across regions. For example, the PIVC, VPS, and VIP seem to carry the strongest spatial tuning, while the MSTd and FEFsem show robust, albeit slightly weaker, responses [(46); also see review by Cheng and Gu (11)].

Temporal and spatial properties are not two completely independent issues. For example, double-peaked neurons typically show one early-peak response in the preferred self-motion direction, while a second late peak appears in the anti-preferred direction. Laurens and et al. developed a three-dimensional (3D) spatiotemporal model based on responses across time and across different self-motion directions to systematically analyze spatiotemporal properties, aiming to characterize how vestibular signals are transmitted from otolith afferents (OAs) through the VN to cortical areas (56). In general, the model fits the experimental data well and reveals that OAs exclusively encode acceleration and jerk signals, while VN and cortical neurons show mixed selectivity for velocity, acceleration, jerk, and position. Thus, the model quantitatively describes to what extent vestibular signals undergo progressive integration as they propagate along the vestibular pathway. Consistent with the experimental observations that the PIVC appears to show the earliest response in the cortex, the model also reveals that neurons in this region have the highest proportion of acceleration signals among all cortical areas examined to date. In contrast, the MSTd is unique in preferentially encoding velocity over acceleration (Figure 1). Finally, other areas encode complex spatiotemporal signals with a rough balance between acceleration and velocity or a slight preference bias toward acceleration. In addition to velocity and acceleration, some cortical regions carry even higher-order temporal signals. For example, Liu and et al. found robust vestibular coding in single neurons in the PCC, and some neurons exhibit strong jerk and position signals (48), potentially related to the hippocampal navigation system.

Characterizing the temporal properties of vestibular signals in each brain area is key to understanding how self-motion is represented and utilized in guiding behavior. The relative weighting of different dynamics may relate to each area’s anatomical connectivity and functional role. For example, some vestibular regions, such as the VPS, PIVC and PCC, project back to the VN according to anatomical evidence (57). Thus, velocity signaling in these pathways may support gaze stabilization and other reflexes with coordinated eye, head, and body movements during natural navigation (58, 59). Similarly, the velocity dominant signals in the MST may also be related to gaze function when the eyes maintain steady pursuit of moving targets when accompanied by self-motion (49). Instead, the acceleration signals in many areas have been shown to be related to heading perception during self-motion (32, 33). In contrast to velocity and acceleration, jerk signals have rarely been studied to date, and their functions remain unclear. Some behavioral studies have examined the impact of jerk signals on subjects’ judgments on discrimination of self-motion strength, yet the conclusions are mixed (60–62). Researchers have indicated using computational modeling that jerk signals exist in some areas of the brain, including the PIVC and PCC (48, 56). Since these areas are within the Papez circuit and are strongly connected to regions involved in unpleasant emotional feelings, such as nausea, dizziness, and pain (63–65), we speculate that the jerk signals may be related to motion sickness during strong and unpredicted self-motion stimuli. Finally, some position signals have been reported in PCC neurons. Interestingly, PCC neurons show a bias for pitch and roll over yaw axes of rotation (48). A previous study also demonstrated that head direction cells in mice maintain fixed azimuthal tuning relative to the horizontal plane, even when the animal’s head orientation changes relative to gravity (66). These properties suggest that position signals in rotation may be critical for anchoring head direction signals to earth-centered coordinates during natural navigation. In summary, vestibular signals contain rich temporal dynamics, and different signals may have distinct functions that require further investigation.

In addition to the vestibular response, many cortical areas are also multisensory and respond to other sensory stimuli, such as optic flow, a cue heavily involved in self-motion perception and path integration during spatial navigation (67). Across regions, the proportion of neurons selective for optic flow is largely varied (Table 1) and is highest in dorsal visual pathways (e.g., the MSTd, FEFsem, and VIP) and lowest in vestibular dominant areas (the VPS, PIVC, and PCC). Unlike the dominant acceleration coding in the peripheral vestibular pathway, visual motion is represented predominantly as velocity signals in the brain (28, 68, 69). Across areas, spatial selectivity for optic flow is MSTd > VIP, FEF > VPS (11, 40, 46), which has an opposite tendency to vestibular flow.

**Table 1:** Proportion of neurons modulated by translational self-motion in different brain areas.

Brain areas	PIVC	VPS	MSTd	VIP	FEFsem	7a	PCC	STP
Vestibular	76.4%	72.3%	64%	49%	71.87%	40%	68%	18.3%
Visual	0	39.8%	98%	69%	85.46%	27%	31%	30.4%

The coexistence of vestibular and visual signals on individual neurons evokes challenges for cue integration when considering the congruency of their temporal and spatial properties. For example, while vestibular signals contain a wealth of temporal information, visual channels predominantly code velocity (70–72). Thus, how different temporal signals are integrated across modalities requires further consideration and investigation (see the following sections). Regarding spatial selectivity, the direction of preference in both modalities can be either congruent or incongruent. Importantly, there is a clear bimodal distribution, such that neurons tend to show highly congruent or nearly opposite heading preferences. This pattern is prevalent across areas, suggesting that it is a general rule in the brain. Interestingly, the VPS is dominated by opposite cells (43). Thus, while congruent cells are thought to be beneficial for multisensory integration (73), the roles of opposite cells are less understood (74).

In addition to the study of vestibular translational motion, some research has been conducted to descriptively analyze how neurons in various vestibular brain regions respond to rotational stimuli. The proportion of responsive neurons to vestibular rotational stimuli varies across different brain regions [MSTd (89%), VPS (75.9%), PCC (59%), VIP (44%), PIVC (49.4%), and 7a (31% for yaw rotation)] (44, 53, 55, 47, 48). Interestingly, the MSTd, which plays a crucial role in visual self-motion perception, has a significantly high proportion of cells responding to vestibular rotational stimuli. Moreover, the DDI is notably higher under vestibular rotation conditions than vestibular translation conditions. Notably, almost all cells under rotational conditions are vestibular-visual opposite cells (55), suggesting that the MSTd does not integrate these signals to produce a robust perception of self-rotation. To date, no conclusive explanation has been obtained from these observations. Due to the absence of behavior-related neural correlation studies, the significance of the proportions of neurons responding to rotation in various brain regions remains further identification in future experiments.

### Reference frame

When observing a spatial variable (e.g., position or velocity), a reference frame can be defined as a specific viewpoint or perspective. The reference frame of the peripheral vestibular system is centered on the head. However, the vestibular reference frame in the central nervous system may be more complex because it must adapt to different functions, particularly when interacting with other sensory signals that are based on different reference frames. In particular, there are several possibilities:

(1) Head-centered reference frame: The vestibular signals remain a head-centered reference frame for heading estimation, while the other signals, for example, eye-centered optic flow, are transformed to head-centered coordinates by integrating with extraretinal signals. Thus, a robust heading representation is maintained under various eye movements during natural navigation.
(2) Eye-centered reference frame: In contrast, vestibular signals may be transformed to an eye-centered coordinate to be aligned with visual signals that typically dominate in many species, such as primates.
(3) Body-centered reference frame: By integrating with other proprioceptive signals, the head-centered vestibular signals could be further transformed into a body-centered reference frame to build a relation between the self and the environment during locomotion and navigation (75).
(4) World-centered reference frame: vestibular signals may ultimately be represented in a world-centered reference frame through double transformation. For example, the “compass” head direction cells, receiving vestibular inputs, are coded in a world-centered coordinate during navigation (76). In the VIP, researchers have discovered world-centered vestibular signals during a world-fixed gaze condition and body-centered signals during a body-fixed gaze condition, indicating that vestibular signals could be either egocentric or allocentric in the posterior parietal cortex (77).

Researchers have measured the spatial tuning functions of single neurons while intentionally manipulating the head, body, and eyes to identify reference frames. For example, Shaikh and et al. measured vestibular tuning in response to different whole-body translation directions while the head was at various fixed positions relative to the trunk (e.g., ±30°). If the spatial tuning functions remain unchanged and are not dependent on different head-on-trunk positions, it suggests that the recorded neuron represents a body-centered coordinate system. In contrast, if the spatial tuning curves exhibit systematic shifts (e.g., ±30°) with the head-on-trunk positions, it indicates that the neuron encodes a head-centered coordinate system. Using a similar approach, previous studies have successfully measured whether vestibular tunings shift with eye, head, or body orientations by varying eye-on-head, head-on-body or body-in-world positions. The central vestibular signals across regions and neurons could be represented in either of the reference frames except the eye-centered reference frame (Table 2; Figure 1).

**Table 2:** Summary of the reference frame of vestibular encoding.

Focus	Classification	Area	Manipulation	Vestibular reference frame	Reference
Head-vs. body-	Brainstem/vestibulo-cerebellum	Rostral VN	Head	Head	(78)
		Rostral FN	Head	Between head and body	(78)
		Rostral FN	Head and body	Between head and body	(79)
Head-vs. eye-	cortex	MSTd	Eye	Head (a modest shift toward eye-centered)	(80)
Head-vs. eye-		FEFsem	Eye	Head	(81)
Head-vs. body-vs. eye-		MSTd	Head and eye	Between eye and head	(75)
Head-vs. body-vs. eye-		PIVC		Between head and body	(75)
Head-vs. body-vs. eye-		VIP		Body	(75)
Body-vs. world-		VIP	Body and gaze	Flexible body and world	(77)

### Vestibular signals in heading discrimination task

As described in the previous sections, vestibular signals are thought to provide idiothetic information for heading perception. Psychophysical experiments have been conducted to test this rationale by using motion platforms or turntables to physically stimulate the vestibular system of humans and nonhuman primate subjects. Subjects are required to report their perceived self-motion direction, for example, heading to the left or right relative to a reference of straight forward. Numerous studies have shown that humans and nonhuman primates can judge their heading directions with a high degree of precision based on vestibular cues alone, particularly monkeys after overwhelming training [Figure 2B; (16, 30, 31, 82–84)]. In particular, the ability of subjects to discriminate heading directions is typically quantified by psychometric functions under a two-alternative forced-choice (2-AFC) experimental paradigm. Participants were found to reliably discriminate deviation from the straight forward direction within a few degrees. For example, macaques can detect deviations of 1°-3.5° based on vestibular cues alone (16), which is close to visual discriminability (31).

To explore neural correlates of vestibular heading perception, researchers have recorded neuronal activity in a number of areas, e.g., the MSTd, that have previously been shown to be modulated by physical motions (26, 37). These modulations in the extrastriate cortex have been shown to arise from a vestibular origin, since labyrinthectomy removes these signals (16). Gu and et al. further recorded vestibular responses while monkeys performed a heading discrimination task on a moving motion platform (16). Aided by receiver operating characteristic (ROC) analysis, researchers can construct neurometric curves to assess an ideal observer’s heading performance based solely on the firing rates of individual neurons. This method, under the framework of signal detection theory (85), allows the direct comparison of heading performance between the ideal observer and the animal. In the MSTd, researchers have found that only a small proportion of neurons exhibit a comparable threshold with the animals, while most of the other neurons show weaker discriminability. This pattern suggests that vestibular heading perception relies on integrating signals across populations of neurons (73) and probably across areas (86). This pattern has also been observed in subsequent studies in other areas, including the VN (87), cerebellum (88), VIP (54), otolith afferents (89), and PIVC (90), suggesting that pooling activity from populations of neurons is a prevalent mechanism in the brain.

In addition to the direct comparison between neuronal and psychophysical sensitivity, simultaneous neural recordings during discrimination tasks allow us to examine neuron-behavior correlations on a trial-by-trial basis. In particular, under identical stimulus conditions, fluctuations in neuronal activities are expected to significantly covary with the behavioral choice [the so-called choice probability or choice correlation, (85)] if the neurons are involved in the decision process. Such significant choice correlations have indeed been discovered in many areas, including the MSTd, VIP, VN, cerebellum and PIVC (16, 54, 88, 90). However, the exact functional implications of choice correlations should be interpreted with caution because numerous factors may lead to significant choice correlations without implying causal link functions, such as noise correlations and top-down feedback signals (91–93). Indeed, although significant choice correlations have been found to be prevalent in the central nervous system, they are essentially lacking in the peripheral afferents, presumably because feedback signals rarely reach the periphery (89).

### Multisensory heading perception

Precise heading perception relies on multisensory cues. While vestibular or visual signals provide useful information about self-motion, they alone could be confounded in many situations (94). Thus, integrating cues can significantly overcome these limits by improving perception.

One significant benefit from cue integration is that it enables finer perceptual sensitivity than that based on either single cue (29). This is indeed the case in vestibular and visual integration: the psychophysical threshold in heading discrimination is reduced when congruent visual and vestibular cues are provided (Figure 2B), and the improvement level is close to the prediction of Bayesian optimal integration theory (31, 82, 95). The underlying neural circuit mediating multisensory heading perception remains unidentified. There are two proposed possibilities that can probably originate back to the weak and strong fusion framework proposed by Landy et al. in multisensory depth perception (96). First, in the early integration model, multisensory information about heading converges somewhere in early/mid stage of sensory cortices before sending to higher level decision areas for evidence accumulation, which is analogous to the strong-fusion framework. In contrast, the late integration model stipulates that unisensory signals are initially transmitted to downstream decision-related regions where integration occurs, reminiscent of weak fusion.

### Temporal and spatial challenges for multisensory integration

Stein et al. performed pioneering studies exploring principles of multisensory integration at the single-neuron level and discovered that efficient multisensory integration requires spatial and temporal coherence (97, 98). Given this, one might expect that visual and vestibular signals would have similar spatiotemporal properties to maximize the cue integration effect. However, recent findings in neurophysiology challenge this intuition.

There are at least two challenges in the spatial domain: the reference frame and spatial congruency of tuning curves. As mentioned above, different sensory modalities originate from different peripheral organs represented in distinct spatial reference frames. One intuition is that the different reference frames should unify somewhere in the central nervous system for cross-cue combination (99), yet this hypothesis is not supported by experimental evidence. In particular, while a head-centered coordinate of visual receptive fields in parietal areas, such as the VIP, has been reported previously [(100); but see (101)], tuning curves modulated by motion directions are mainly coded in an eye-centered reference frame in a number of cortices, including the MSTd (80, 81), VIP (41), V6 (102), and FEFsem (81). In contrast, vestibular signals are mainly based on a head-to body-centered reference frame in the MSTd, VIP, and FEFsem (77, 80, 81), although some neurons encode a head-centered coordinate that is modestly shifted to an eye-centered coordinate in the MSTd (80). Thus, most of the experimental findings in neurophysiology indicate that the visual and vestibular reference frames for self-motion perception are largely separated. While computational modeling works demonstrate that population readout of eye-centered visual and extraretinal signals could recover head-centered self-motion information, evidence of such head-centered coding in single neurons in sensory cortices remains lacking.

Another challenge is the presence of a large population of neurons that prefer nearly opposite self-motion directions indicated by vestibular and optic flow cues. Neurons representing conflict information exist across numerous sensory cortices [(43, 46); also see review by Cheng and Gu (11)] and sensory modalities, for example, horizontal disparity and motion parallax cues indicating depth (103), suggesting that this pattern is prevalent in the brain. Unlike congruent cells, neuronal tuning in opposite cells would be reduced when exposed to congruent sensory inputs. Thus, the opposite cells are unlikely to contribute to improved heading precision during cue combination (31, 73). The exact functions of this type of neuron require future studies with some proposed functions, including (1) distinguishing object motion and self-motion (74, 103, 104) and (2) serving as computational units for causal inference between multisensory integration and segregation (105).

Regarding the temporal domain, we have mentioned that visual signals are velocity-dominant in sensory cortices, whereas vestibular signals show varied temporal components in the central nervous system. According to the temporal-congruency principle in multisensory integration, it is expected that congruent visual and vestibular signals (i.e., velocity) should facilitate cue integration. The MSTd seems to be an ideal substrate. In particular, the MSTd neurons largely exhibit congruent visual and vestibular temporal dynamics, that is, velocity (28, 31). During the cue-combined condition, responses across the modalities are well-aligned and subsequently facilitate enhancement.

**Figure 3:**
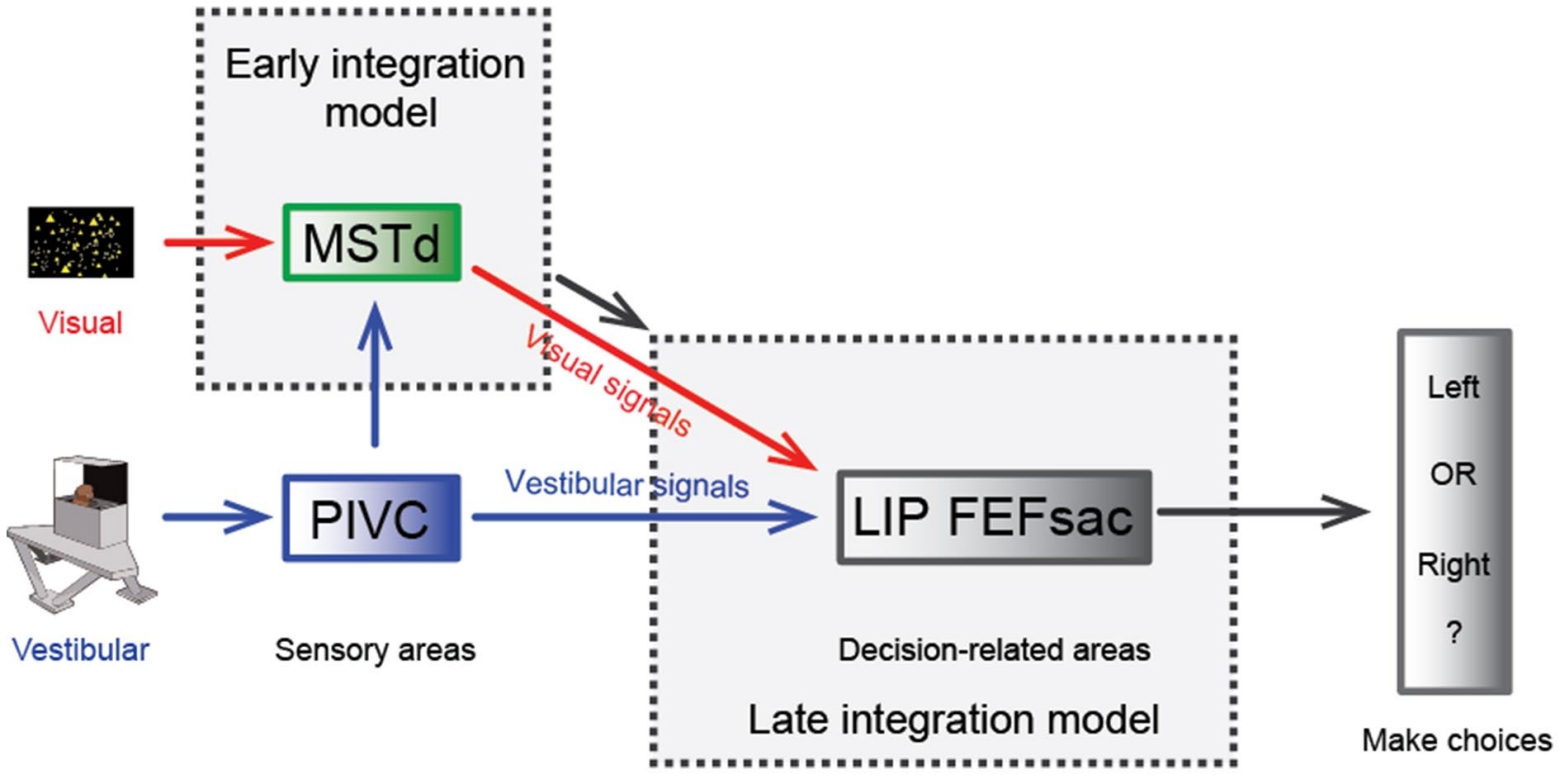
Hypothesis of multisensory integration mechanisms. Schematic diagram of the hypothesis of the multisensory integration mechanism. In the early integration model, vestibular (from the PIVC) and optic flow signals are first integrated in the sensory area, the MSTd, then transmitted to high-level decision-making areas. In the late integration model, the two heading signals do not converge until they are transmitted to decision-related areas, such as the LIP or FEFsac.

Hence, a specific population of neurons in the MSTd appears to meet both the spatial and temporal congruent principle in multisensory integration and thus may be beneficial for multisensory heading perception. However, recent experimental findings challenge this view. First, causal link experiments with electrical stimulation and chemical inactivation applied in MSTd fail to produce significant effects on the animals’ judgment of heading direction under vestibular conditions, while the effect is highly significant under visual conditions [Figure 3; (106)]. As a comparison, inactivating the PIVC significantly impairs the animals’ vestibular performance [Figure 3; (107)]. Thus, the MSTd seems to play a critical role in visual but not vestibular heading perception. Second, researchers have found that in a heading discrimination task based on a reaction time version (108), a human subject’s performance in the combined vestibular, visual and cue conditions is consistent with a model in which the brain accumulates vestibular acceleration and visual velocity evidence. Later, when macaques performed the multisensory heading discrimination task, Hou et al. (32) and Zheng et al. (33) recorded single neurons in the LIP and the saccade region of the FEF (FEFsac), two brain areas thought to be involved in perceptual decision-making tasks (109). They found that populations of neurons in both regions exhibit similar temporal dynamics. In particular, under the vestibular-only condition, ramping activities are more aligned with a process when momentary acceleration evidence is accumulated. In contrast, visual response dynamics are more aligned with the accumulation of velocity signals. Comparing the two stimulus conditions, vestibular ramping activity leads the visual by a few hundred milliseconds, which roughly corresponds to the lag between the acceleration and velocity peak in the motion profile used in the experiments. This result was further substantiated through causal inference experiments in which the researchers systematically manipulated the bandwidth of the Gaussian stimulus profile. By modulating the acceleration peak time while keeping the velocity peak constant, they could shift the acceleration profiles independently of velocity. Under these conditions, they observed that the ramping visual neuronal responses were unchanged, whereas vestibular response timing shifted in accordance with the changes in acceleration peak (32). Thus, either human psychophysical experiments based on reaction time tasks or monkey physiological experiments indicate that the brain may employ incongruent vestibular and visual temporal signals for heading judgment. This finding suggests that another possible model (the late integration model) is more likely to be true (Figure 3).

**Figure 4:**
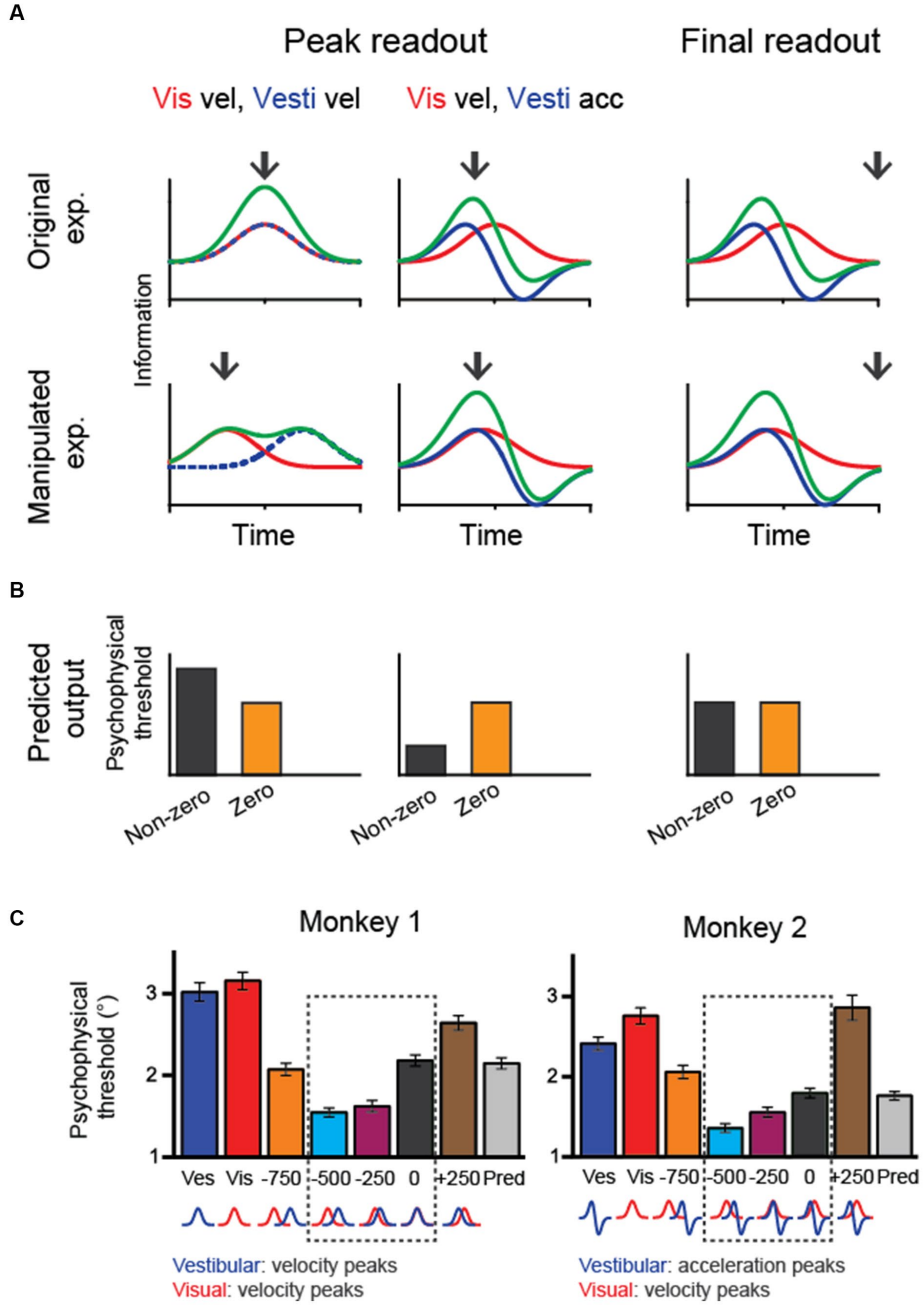
Manipulating temporal offset in the visuovestibular inputs and hypothetical behavioral outputs. **(A)** Fisher information in vestibular, visual and combined conditions when the temporal offset between the two heading cues is zero (up row) or nonzero (bottom row). Black arrows indicate hypothetical readout time. **(B)** Predicted psychophysical threshold for each model output. For the final-readout model, the results are the same for either the temporal-congruent or incongruent model; thus, only one case is shown (temporal incongruent model). **(C)** The performance of two monkeys shows improved heading performance during cue-combined conditions when the visual input is artificially adjusted to lead the vestibule by 250–500 ms. At the bottom of the plots, corresponding velocity and acceleration profiles of visual and vestibular cues are shown for each temporal-offset condition. Redrawn with permission from Zheng et al. (33).

In macaque experiments, researchers have used a fixed duration paradigm in which the animals experience heading stimuli for a fixed amount of time (e.g., 2 s). Thus, it is unclear how downstream areas reconcile the discrepancy between vestibular and visual heading cues in the temporal domain. A few different strategies could integrate sensory evidence over time (Figure 4). One is to keep collecting sensory information up to the end of a trial (termed the “final readout” strategy). Alternatively, information may be continuously collected until the moment when upstream areas provide maximal information (“peak readout” strategy). Considering the two visuo-vestibular temporal integration models as aforementioned (vestibular acceleration and visual velocity versus velocity for both modalities), there are a few outcomes in the behavioral performance during the bimodal stimulus condition based on all possible combinations. First, for the “final-readout” strategy, either the visuo-vestibular temporal congruent or incongruent model would produce identical behavior because all information is ultimately collected by reaching the same level at the end of a trial. For the “peak readout” strategy, however, information is enhanced more in the temporal-congruent model at the moment when both cues reach their peak response, unlike in the temporal-incongruent model in which sensory cues are not aligned. Thus, the key question is which method the brain uses to perform the multisensory heading task.

To disentangle these possibilities, researchers have systematically varied the temporal offset between the visual and vestibular inputs and examined the behavioral performance. The trick is to artificially align the visual velocity peak in the motion profile with the vestibular acceleration peak by leading the visual input for a few hundred milliseconds. Interestingly, monkeys demonstrated better performance under this manipulation [Figure 4A; (33)]. Such a result first rules out the “final readout” strategy that predicts identical performance regardless of temporal offset between single cues. Second, the result is consistent with the prediction from the “peak readout” strategy with the visuo-vestibular temporal incongruent model. Thus, the behavioral performance under temporal-offset manipulated experiments suggests that the brain used vestibular acceleration and visual velocity signals to perform the heading task. Importantly, the behavioral pattern is consistent with neural activities simultaneously recorded in the LIP and FEFsac. In particular, information capacity in the population of sensory-motor association neurons is highest during the manipulation of visuo-vestibular sensory inputs, precisely explaining the behavior.

Result from the temporal offset manipulation experiment could also fit into the causal inference model (34). In particular, the brain still integrates the vestibular and visual signals when the two stimuli inputs are offset within a small amount (e.g., <500 ms), as reflected by the reduced psychophysical threshold compared to the condition when no offset is introduced. However, when the offset is large, for example, 750 ms, psychophysical threshold is instead increasing, suggesting that the brain is not integrating the two cues any more, but rather rely on one of the two cues.

## Discussion

In summary, the vestibular system is fundamental for self-motion perception because of its unique spatiotemporal properties and its tight entanglement with other sensory channels in the central nervous system. Decades of research on single-unit electrophysiological recordings in animal models, such as macaques, have provided valuable data with high spatiotemporal resolutions in identifying cortical areas that are involved in processing vestibular signals. Combined with behavioral tasks and neural activity manipulations, both correlation and causality could be identified for each region of interest (ROI) with cognitive functions, such as self-motion perception. Further aided by new techniques, including large-scale recordings and imaging methods, scientists could identify more brain-wide regions associated with self-motion and ultimately construct a vestibular neural network for self-motion perception. In addition, there are several issues that need to be considered for future research:

(1) Based on the experimental findings, researchers have proposed a 3D spatiotemporal model to quantify the complex temporal and spatial coding for vestibular signals (48, 56). The models overall capture the data well; however, the high dimensionality and nonlinearity in this model make it prone to overfitting. Further work should optimize the model parametrization and design more sophisticated experimental conditions to better disentangle different signaling components. In particular, previous experiments typically use Gaussian velocity profiles with biphasic acceleration profiles. In this case, the distinction of different signals (velocity, acceleration and jerk) could be difficult because the main feature of the peak time in each component may not differ enough. Introducing more features should help differentiation. For example, in de Winkel’s work (60), by manipulating the peak amplitude of acceleration and jerk stimuli at different levels, the contributions of different signals to the perception of self-motion intensity could be better distinguished. Similar ideas could be applied to neurophysiology.
(2) The complex temporal dynamic properties suggest multiple possible roles for different vestibular signals in self-motion, locomotion and navigation. Recent evidence indicates that sensory-motor transformation areas, including the parietal and frontal lobes, accumulate acceleration instead of velocity vestibular signals for heading judgments (32, 33). Vestibular signals indicating self-motion information are typically considered an important source for vector-based navigation. In addition to the cerebral cortex, vestibular signals are also thought to be transmitted to the hippocampal areas through the ADN (5). Theoretical models and lesion experiments indicate that head direction cells originate from semicircular canals that provide input of angular velocity signals, and grid cells receive linear velocity signals that originate from the otolith system (110–112). However, the exact cortical-to-hippocampus pathways that convey vestibular signals remain unclear. Future studies with new techniques, including large-scale recordings and imaging methods, may identify these connections.
(3) Self-motion perception relies on vestibular cues during active and passive movements. Previous studies on self-motion mainly used instruments such as rotating chairs, motion platforms, or centrifuges to generate vestibular stimuli in passive motion conditions. During active motion, however, vestibular signals that match predictions of motor command signals are typically suppressed to avoid reflex and consequently enable intended actions (113). This is indeed found in neurophysiology: while vestibular coding is comparable in the vestibular afferents in active and passive conditions (114, 115), responses are largely attenuated in the central nervous system, including the VN, cerebellum and thalamus, during active motion conditions (116–118). This raises an obvious question of how vestibular signals are then utilized for direction and distance judgments in spatial navigation and cognition during active self-motion. One possibility is that vestibular information from vestibular afferents is retained in alternative pathways. Indeed, it has been found that the activities of PIVC neurons do not differ in their response to active and passive motion (119). In the VIP, researchers found that some neurons change directional tuning preference or firing rates during active self-motion, while the other neurons do not change (120). Thus, vestibular coding during active self-motion in other cortical areas requires further examination in future studies.
(4) Although the encoding characteristics of the vestibular system have been extensively studied under laboratory conditions, the vestibular stimuli that can be provided under such conditions are limited within a finite range. Carriot et al. (121), for the first time, measured the statistical characteristics of vestibular input received by human subjects during their natural movements in daily activities. Subsequently, similar statistical analyzes were conducted in macaque monkeys and rodents (122). Additionally, the intensity of these motions often falls below the level achievable during unrestricted movement. Interestingly, it has been observed that the vestibular system exhibits adaptive encoding during natural movement (19). This implies that relying exclusively on limited artificial motion stimuli might not adequately capture the encoding patterns that occur under naturalistic stimuli. Similarly, the optic flow stimuli received by the retina are often more intricate during natural navigation. Employing advanced eye, head, and body tracking technologies allows for the concurrent evaluation of eye movements, head motions, and gait. This process aids in reconstructing the retinal optic flow experienced during natural motion, thus enabling the depiction of fundamental features in the statistics of human visual motion during natural activities (123, 124). While accurate retinal motion statistics have been obtained, further electrophysiological research is needed to explore the responsive properties of neurons to visual motion during natural movements.
(5) This review largely focused on translation stimuli because considerable work has been conducted on how vestibular signals originating from the otolith system contribute to linear self-motion (heading) perception. In addition to the translation signals, rotation signals originating from the semicircular canal system are also important for self-motion perception. In fact, many cortical regions encode both translation and rotation self-motion (28, 40, 44, 47, 48), and the two signals frequently converge into individual neurons (125–127). Compared to translation, the contribution of rotation signals to behavioral tasks has been less studied (13, 15, 128–130). In these psychophysical studies, primarily through psychophysical experiments, researchers investigated the threshold at which human participants detected rotational motion in darkness and determined the significance of vestibular signals in rotational perceptual ability. In comparison, studies involving neural recordings during rotation tasks are rare. Only one study addressed neuronal data; in a rotation discrimination task, Garcia and et al. showed that unlike in translation tasks, VN neurons lack significant choice correlations with behavioral performance (131). This finding suggests that the brain may handle different motion types in different ways. A key difference between the two motion systems is that while the otolith organs mainly transfer acceleration signals, the output of canals mainly carry velocity signals due to its mechanical dynamics (50, 132). Thus, while decision-related neurons mainly accumulate acceleration signals for linear heading perception, whether more velocity components are accumulated by the decision-related neurons for rotation perception needs to be examined in future experiments. Finally, curved motion, which comprises translation and rotation, is another important type of self-motion in daily life (128, 133–136). Neurophysiological studies have shown that a population of neurons in the VN (125, 127) and cerebral cortex (126) prefer curved motion stimuli, suggesting that these neurons integrate inputs from both otolith and semicircular canals simultaneously. The mixed coding of translation and rotation signals in individual neurons may support the hypothesis that these neurons could mediate curvilinear self-motion perception (126). Alternatively, either translation or rotation signals may be flexibly decoded from the population of these neurons according to the needs presented by the task. For example, in a recent study, researchers used an electrical microstimulation technique to artificially activate a group of visual neurons that encode both translation and roll-rotation. It has been shown that microstimulation significantly biases the animals’ perceived translation direction in linear heading discrimination trials and biases the perceived roll direction in rotation discrimination trials (137). However, whether the same flexible decoding strategy applies in vestibular coding remains to be examined in future studies.

## Author contributions

BL: Writing – original draft, Writing – review & editing. JS: Writing – original draft, Writing – review & editing. YG: Conceptualization, Funding acquisition, Project administration, Validation, Writing – review & editing.

## Funding

The author(s) declare financial support was received for the research, authorship, and/or publication of this article. This work was supported by grants from the Strategic Priority Research Program of CAS (XDB32070000) and the Shanghai Municipal Science and Technology Major Project (2021SHZDZX) to YG.

## Conflict of interest

The authors declare that the research was conducted in the absence of any commercial or financial relationships that could be construed as a potential conflict of interest.

## Publisher’s note

All claims expressed in this article are solely those of the authors and do not necessarily represent those of their affiliated organizations, or those of the publisher, the editors and the reviewers. Any product that may be evaluated in this article, or claim that may be made by its manufacturer, is not guaranteed or endorsed by the publisher.
